# The application value of MRI in the diagnosis of subclinical inflammation in patients with rheumatoid arthritis in remission

**DOI:** 10.1186/s13018-018-0866-2

**Published:** 2018-07-03

**Authors:** Huimei Zhang, Huajun Xu, Shifang Chen, Xinfeng Mao

**Affiliations:** 1 Department of Radiology, Huzhou Central Hospital, Huzhou, 313000 Zhejiang People’s Republic of China; 2 Department of Ultrasound, Huzhou Central Hospital, No. 198 Hongqi Road of Wuxing District, Huzhou, 313000 Zhejiang People’s Republic of China; 3 Department of Rheumatology and Immunology, Huzhou Central Hospital, Huzhou, 313000 Zhejiang People’s Republic of China

**Keywords:** Bone marrow edema, Early rheumatoid arthritis, MRI, Remission

## Abstract

**Background:**

To explore the value of MRI in the diagnosis of subclinical inflammation in patients with early rheumatoid arthritis (RA) in remission and to predict the radiographic progression.

**Methods:**

A total of 76 of 156 patients with early RA in remission at 1 year and with available magnetic resonance imaging (MRI) data at baseline and at 12 months were included. Complete clinical and laboratory evaluations were conducted for the patients. MRI images were assessed according to the Rheumatoid Arthritis Magnetic Resonance Imaging Scoring (RAMRIS) system. Progression of bone erosions was defined as an increase of 1 or more units in annual RAMRIS score for erosions compared to baseline.

**Results:**

At 1 year, the majority of patients with RA in sustained remission showed some inflammatory activity on MRI (43.4% synovitis, 39.5% bone marrow edema (BME), and 9.2% tenosynovitis), and 25 of the 76 patients (32.9%) showed MRI progression of bone erosions. A significant difference was observed in MRI BME and bone erosion at 1 year, with higher mean score in patients with progression compared to non-progression of erosions (BME, 4.8 ± 3.6 vs 3.1 ± 2.1, *P* = 0.01; bone erosion, 13.5 ± 9.6 vs 4.4 ± 3.6, *P* < 0.001).

**Conclusion:**

Persistent subclinical inflammations were shown in patients with sustained remission; BME in MRI may be a strong predictor of future radiographic progression of bone erosions in patients with persistent clinical remission.

## Background

Rheumatoid arthritis (RA) is a common chronic autoimmune disease characterized by aggressive arthritis, causing pain, stiffness, swelling, and limited motion [1, 2]. The inflammatory process of RA can result in progressive cartilage degradation with synovial hyperplasia, high levels of pro-inflammatory mediators, and change in underlying bone with erosions [3]. Early diagnosis and prompt initiation of treatment are needed to improve physical function and reduce disability and joint destruction in patients with RA [4, 5]. Currently, the chief aim of treatment for patients with RA is to achieve a state of disease remission or low levels of disease activity and stop progression of structural damage [6]. However, RA still has different degrees of subclinical arthritis in the clinical remission period, which is one of the important factors in the development of RA disease [7, 8]. Therefore, how to find subclinical arthritis to help improve clinical treatment plan, effectively delay the occurrence of bone destruction, and improve the quality of life of patients is of great significance.

The new methods to diagnose and monitor RA have evolved. Imaging of inflammatory activity becomes more and more important among the available modalities [9]. In particular, magnetic resonance imaging (MRI) is a more promising tool than clinical examination or conventional radiography, due to it performed on high field units and could offer the opportunity for detecting inflammation and joint erosions [10, 11]. Many researches have reported that MRI could directly visualize and assess synovitis, bone marrow edema (BME), synovial thickening, cartilage destruction, tenosynovitis, and bone erosion for patients in remission or low disease activity state, in which the MRI subclinical inflammation perhaps explains the structural progression on radiography [4, 5, 10–12].

The aim of this study was to explore the value of MRI in the diagnosis of subclinical inflammation (synovitis, BME, and tenosynovitis with the progression of erosions) and to predict the radiographic progression in patients with early RA in remission at 1 year.

## Methods

### Patients

A total of 156 patients with early RA, who visited the Department of Rheumatology and Immunology of Huzhou Central Hospital from January 2015 to June 2017, were enrolled. This study was conducted with the approval from the Ethics Committee of Huzhou Central Hospital. Written informed consent was obtained from all participants. After admission, routine medical history, physical, laboratory, and bilateral wrist MRI examination were performed in all patients. Then, all patients should be treated initially with disease-modifying antirheumatic drugs (DMARD). At study entry, all patients fulfilled the 2010 American College of Rheumatology/European League Against Rheumatism (ACR/EULAR) criteria for RA and had active disease of ≤ 12 months’ duration with one or more swollen joints. All patients excluded were aged < 18 years, had history of joint trauma and other rheumatism, had taken glucocorticoid in the past 1 month, previously with DMARD, and had active disease of > 12 months’ duration. Lastly, a total of 76 patients were included in this retrospective study.

### Demographic and clinical assessment

Complete clinical and laboratory evaluations were conducted for the patients. Age, sex, disease duration, the erythrocyte sedimentation rate (ESR), disease activity score (DAS), C-reactive protein (CRP), rheumatoid Factor (RF), and anticyclic citrullinated peptide antibody (ACPA) values were recorded. Standardized joint counts, including tender joints (TJCs) and swollen joints (SJCs), were recorded. Clinical characteristics were assessed at baseline and at 3, 6, and 12 months. The period of disease activity was defined as a disease state of DAS28 > 2.6. The clinical remission was defined as a disease state of DAS28 ≤ 2.6. Persistent clinical remission was defined as fulfilling this definition for a period of least 6 months without flares or treatment changes before completing 1-year follow-up. Only patients who fulfilled persistent clinical remission were included in the analysis of bilateral wrist MRI examination in this study.

### MRI procedures

MR imaging was performed by using a Siemens Trio 3.0-T Magnetom Symphony imager (Siemens, Erlangen, Germany) with special coil of wrist joint. The following sequences were acquired before contrast agent injection: Spin echo (SE), T1 weighted imaging (T1WI), coronal plane (500 ms repetition time (TR); 22 ms echo time (TE), 256 × 256 matrix, 3 mm section thickness, 0.3 mm intersection gap), and Fast SE T2 weighted imaging (T2WI) coronal and axial plane (3000 ms TR; 85 ms TE, 256 × 256 matrix, 3 mm section thickness, 0.3 mm intersection gap). Then, gadopentate dimeglumine pentaacetic acid (Gd-DTPA, Magnevist, Schering, Berlin, Germany) was injected intravenously at a dose of 469.01 mg/ml × 15 ml. After intravenous contrast injection, contrast-enhanced fat suppression-fast spin echo FS-FSE-T1WI transverse (650 ms TR; 22 ms TE, 256 × 256 matrix, 3-mm section thickness, 250× 100 mm field of view (FOV)) and coronal plane were obtained (650 ms TR; 22 ms TE, 256 × 256 matrix, 3 mm section thickness, 230 mm × 250 mm FOV). The fat suppression technique applied spectral fat saturation inversion recovery (SPIR). The scanning range of the transverse section ranged from the proximal end of the ulnar and radial joint to the distal interphalangeal joint. The coronal continuous scans were performed from the palmar to the dorsal, including the wrist, metacarpophalangeal (MCP), and proximal interphalangeal (PIP) joints. Enhanced scan was completed within 5 min after injection of contrast agent.

### MRI evaluation

The MR image sets of bilateral wrist joint (including the wrist, MCP, and PIP joints) were assessed for synovitis, tenosynovitis, BME, and bone erosion according to the OMERACT (Outcome Measures in Rheumatology) MRI scoring system (RAMRIS) [13, 14]. Briefly, synovitis is scored from 0 to 3 (none, mild, moderate, severe), tenosynovitis is scored from 0 to 3 (none, mild, thickness of tendon sheath < 1.5 mm; moderate, 1.5 mm ≤ thickness of tendon sheath < 3 mm; severe, thickness of tendon sheath > 3 mm), BME is scored from 0 to 3 based on the volume of edema (0%, 1–33%, 34–66%, 67–100% of edematous bone), and bone erosions are scored from 0 to 10 based on the proportion of eroded bone (from 0% to 91–100%) [13]. In addition, we defined progression of joint erosion as an increase of 1 or more units in annual RAMRIS score for erosions compared to baseline [4]. According to this definition, patients were placed into two groups: “progressors” (P) and “nonprogressors” (nP) at 1 year.

All MRIs were independently interpreted and consensus by two rheumatologists, who were trained in the evaluation of MR images of RA joints and blinded to the clinical data. The intraclass correlation coefficients (ICC) for intrareader and interreader reliability for a single measure and change were calculated using a two-way random effect model. Single-measure ICC and average-measure ICC for status and change scores were calculated and were given as medians and ranges. Agreement was considered good if ICC were > 0.60 and very good at > 0.80 [4]. In this study, within-reader ICCs for the total MRI inflammation score were 0.99 and 0.93; the between-reader ICC was 0.87.

### Statistical analysis

Statistical analyses were performed using the SAS 9.3 statistical software. Qualitative data were expressed as percentage, as $\overline{x}$ ± s when it was in accordance with the normal distribution, and as median (M) ± quartile (Q) when it was in accordance with skewed distribution. *T* test or *χ*^2^ was used to compare the differences between groups. For all statistical values, *P* < 0.05 was considered to indicate a statistically significant difference.

## Result

### Patient characteristics

Seventy-six patients with early RA (62 females and 14 males, aged 42.6 ± 12.2 years, disease duration of 8.3 ± 4.2 months) were included in this retrospective study. One year after treatment, the MRI of RA remission showed subclinical arthritis, including synovitis (33/76, 43.4%), BME (30/76, 39.5%), and tenosynovitis (7/76, 9.2%). In addition, there were 25 cases (25/76, 32.9%) that had bone erosion radiographic progression.

### Comparison of activity and remission period of early RA

RAMRIS score for synovitis, BME, and tenosynovitis showed a significant reduction in patients in clinical remission at 1 year, while the mean RAMRIS score for bone erosions was similar (6.4 ± 5.6 vs 7.1 ± 6.5, *P* = 0.31) at baseline and at 1 year (Table 1). In addition, as Table 1 shows, there were significant differences in terms of TJC28, SJC28, DAS28, ESR, and CRP between RA remission and disease activity period. However, there were no significant differences in terms of RF (77.6 vs 69.7%, *P* = 0.27) and ACPA levels (82.9 vs 77.6%, *P* = 0.42) between RA remission and disease activity period (*P* > 0.05).

**Table 1:** Clinical and MRI variables of patients in activity period and remission at 1 year

Characteristics, *n* = 76	Baseline	1 year	*P* value
Clinical variables			
TJC28	4.2 ± 3.1	0	< 0.001
SJC28	3.5 ± 2.6	0	< 0.001
DAS28	4.2 ± 1.3	1.9 ± 0.5	< 0.001
ESR	32.5 ± 12.6	14.6 ± 6.1	< 0.001
CRP	15.8 ± 5.4	6.3 ± 3.2	< 0.001
RF	59/76 (77.6%)	53/76 (69.7%)	0.27
CCP	63/76 (82.9%)	59/76 (77.6%)	0.42
MRI variables			
Synovitis	6.1 ± 3.8	4.0 ± 2.4	< 0.001
BME	5.9 ± 4.6	2.9 ± 2.2	< 0.001
Tenosynovitis	4.9 ± 3.4	1.6 ± 1.1	< 0.001
Bone erosion	6.4 ± 5.6	7.1 ± 6.5	0.31

*ESR* erythrocyte sedimentation rate, *DAS* disease activity score, *CRP* the C-reactive protein, *RF* rheumatoid Factor, *ACPA* anticyclic citrullinated peptide antibody, *TJCs* tender joints, *SJCs* swollen joints, *BME* bone marrow edema

### Comparison of P and nP at baseline

At entry into the study, no significant difference was observed between the P and nP groups of patients in terms of synovitis, tenosynovitis, bone erosion, TJC28, SJC28, DAS28, ESR, RF, and ACPA values (all *P* > 0.05). However, the 25 patients showing progression of bone erosions showed significantly higher values of CRP (P vs nP, 21.6 ± 10.2 vs 15.6 ± 8.8; *P* = 0.01) and MRI BME (P vs nP, 8.3 ± 6.4 vs 3.7 ± 3.1; *P* < 0.001) (Table 2) than nP group.

**Table 2:** Baseline and annual clinical, laboratory, and MRI characteristics according to MRI evidence of erosion progression at 1 year in patients with RA in remission

Characteristics	Baseline		*P*	1 Year		*P*
	Progression (*n* = 25)	No progression (*n* = 51)		Progression (*n* = 25)	No progression, (*n* = 51)	
Clinical variables						
TJC28	6.3 ± 5.8	4.2 ± 3.6	0.06	0	0	–
SJC28	3.6 ± 2.1	2.9 ± 1.8	0.14	0	0	–
DAS28	4.5 ± 2.5	3.6 ± 2.1	0.11	1.9 ± 0.8	1.6 ± 0.7	0.10
ESR	38.3 ± 18.9	29.8 ± 17.6	0.06	12.1 ± 8.2	14.5 ± 9.4	0.29
CRP	21.6 ± 10.2	15.6 ± 8.8	0.01	7.3 ± 4.6	6.5 ± 5.2	0.52
RF	19/25 (76%)	40/51 (78.4%)	0.22	16/25 (64%)	37/51 (72.5%)	0.15
CCP	21/25 (84.0%)	42/51 (82.3%)	0.25	20/25 (80%)	39/51 (76.5%)	0.23
MRI						
Synovitis	6.3 ± 3.4	5.9 ± 2.9	0.60	4.6 ± 2.0	3.7 ± 2.6	0.13
Tenosynovitis	5.2 ± 3.8	4.6 ± 3.6	0.51	2.1 ± 0.9	1.7 ± 0.8	0.06
BME	8.3 ± 6.4	3.7 ± 3.1	< 0.001	4.8 ± 3.6	3.1 ± 2.1	0.01
Bone erosion	9.9 ± 8.6	8.1 ± 6.9	0.34	13.5 ± 9.6	4.4 ± 3.6	< 0.001

*ESR* erythrocyte sedimentation rate, *DAS*, disease activity score, *CRP* the C-reactive protein, *RF* rheumatoid Factor, *ACPA* anticyclic citrullinated peptide antibody, *TJCs* Tender Joints, *SJCs* Swollen Joints, *BME* bone marrow edema

### Comparison of P and nP at 1 year

The mean values for the DAS28, ESR, CRP, RF, and ACPA values had no significant difference between P and nP groups of patients with sustained remission at 1 year. However, a significant difference was observed in MRI BME and bone erosion, with a higher mean score in P patients compared to nP (BME, 4.8 ± 3.6 vs 3.1 ± 2.1, *P* = 0.01; bone erosion, 13.5 ± 9.6 vs 4.4 ± 3.6, *P* < 0.001) (Table 2).

## Discussion

Early diagnosis and treatment of RA is necessary to prevent the progression of bone destruction. Previously, the examination modalities used for the assessment of disease activity and structural joint damage included clinical examination, biochemical assessment, composite disease activity scores, and conventional radiography [4]. However, they are not sufficiently sensitive or specific, especially in early RA. Currently, many reports support that MRI is more sensitive than clinical examination for detecting joint inflammation, especially subclinical inflammation; moreover, it has shown superior sensitivity compared to conventional radiography for detecting erosions in RA [4, 5, 10–12]. Comparison of findings on X-ray examination and MRI showed that MRI detects more erosions than plain film radiography [15]. In addition, ultrasound examination is economical and convenient; however, it reduces the reliability of the assessment of the disease activity, due to high sensitivity to synovitis and early bone erosion, is highly dependent on the doctor’s experience, and cannot evaluate the bone marrow edema [16]. Therefore, MRI is the most reliable imaging examination of the early RA [17].

The pathological basis of RA is synovial hyperplasia, synovitis, and bone erosion (active intra-articular inflammation leads to synovial hyperplasia and synovial pannus causing bone erosion) [2]. Effective anti-rheumatoid treatment could reduce disease activity, reduce intra-articular inflammation, and bring disease into remission stage. This study found that TJC28, SJC28, DAS28, ESR, CRP, synovitis, tenosynovitis, and BME were significantly lower in RA remission period than that in the active period. However, in the RA remission period, there are still some subclinical arthritis, including synovitis (*n* = 33, 43.4%) and BME (*n* = 30, 39.5%), which had the risk of bone erosion and could promote the progression of the disease. Early diagnosis and treatment of RA are therefore important for preventing joint destruction [18].

Effective DMARD can reduce disease activity, keep patients at low activity level, and prolong remission stage. However, as a primary autoimmune disease, because of the unknown etiology, it is still lacking effective drugs to completely cure it, and the RA clinical remission period still has the potential risk of the progress of bone erosion. In this study, 25 patients (25/76, 32.9%) were found to have remission of RA. After 1 year, the score of MRI bone erosion was increased, and the progress of the disease was confirmed by imaging. In this study, 25 cases (32.9%) of RA remission patients were found. After 1 year, bone erosion score was increased, and the progression of disease was confirmed by MRI. Therefore, the subclinical inflammation in joints of remission RA were found by MRI, which could improve the clinical treatment plan, further reduce the degree of disease activity, inhibit the progress of bone erosion, and improve the therapeutic effect.

BME and synovitis, frequently occurred together, are common subclinical intra-articular inflammation of RA. Synovitis is caused by the inflammatory factors that were produced by RA autoimmune stimulation. On the one hand, BME is related to the infiltration of inflammatory cells into subchondral cortical bone. On the other hand, the high expression of nuclear factor κB receptor causes BME, which stimulate osteoclasts and other bone autoimmune reactions, causing bone erosion. Previously, researchers had different views on whether BME or synovitis could predict RA disease progression [19]. Recently, BME has been reported to represent the frequent independent predictor of radiographic progression in early and active RA [20]. In this study, we indicated that BME was closely related to bone erosion, at early diagnosis of RA; the higher the BME score, the heavier the inflammation in the joint, and the higher the CRP value, the higher the risk of bone erosion occurred in one year. Therefore, we concluded that the BME could predict the future progress of the disease in the RA patients, which was similar to previous studies, such as Mao et al. [21] and McQueen et al. [19]. Therefore, how to effectively diagnose subclinical arthritis in remission stage and to further reduce bone marrow edema is of great significance for delaying the progress of RA disease, prolonging remission period, and inhibiting the risk of bone erosion.

This study has several limitations. Firstly, the standard of disease relief was based on DAS28, and no energy Doppler ultrasound was used to evaluate the disease activity in the remission stage. Secondly, the limited number of patients analyzed could account for some of the differences from the results found in other studies related to progression of erosions. Therefore, in the future, a larger sample is furtherly needed to confirm the study through ROC curve analysis.

## Conclusion

In general, persistent subclinical inflammations were shown in patients with sustained remission; BME in MRI may be a strong predictor of future radiographic progression of bone erosions in patients with persistent clinical remission.
